# c-MYC drives histone demethylase PHF8 during neuroendocrine differentiation and in castration-resistant prostate cancer

**DOI:** 10.18632/oncotarget.12310

**Published:** 2016-09-28

**Authors:** Peterson Kariuki Maina, Peng Shao, Qi Liu, Ladan Fazli, Scott Tyler, Moman Nasir, Xuesen Dong, Hank Heng Qi

**Affiliations:** ^1^ Department of Anatomy and Cell Biology, Carver College of Medicine, University of Iowa, Iowa City, IA 52246, USA; ^2^ Vancouver Prostate Center, Department of Urology Sciences, University of British Columbia, Vancouver, BC V6H 3Z6, Canada; ^3^ Department of Health and Human Physiology, College of Liberal Arts and Sciences, University of Iowa, Iowa City, IA 52242, USA

**Keywords:** prostate cancer, NED, CRPC, PHF8, c-MYC

## Abstract

Epigenetic factors play critical roles in prostate cancer (PCa) development. However, how they contribute to neuroendocrine differentiation (NED) and castration-resistant PCa (CRPC) is not fully understood. Using bioinformatics and biochemical approaches to analyze cell-based models of NED and CRPC, we found a cluster of epigenetic factors whose expression is downregulated during NED and upregulated in CRPC (i.e. follow a Down-Up pattern). Two histone demethylases within this cluster, PHF8 and KDM3A, are post-transcriptionally regulated by c-MYC through miR-22, which targets both *PHF8* and *KDM3A*. We also found that the c-MYC/miR-22/PHF8 axis is downstream of androgen receptor (AR) signaling in CRPC cells. The co-expression of PHF8 with AR in clinical CRPC samples, normal mouse prostate, and adenocarcinomas of the prostate during PCa progression in a transgenic (TRAMP) mouse model supports the connection between PHF8 and AR. Knockdown of PHF8 impedes cell cycle progression in CRPC cells and has more profound effects on their growth than on the parental LNCaP cell line. Furthermore, PHF8 knockdown sensitizes LNCaP-Abl cells to the AR antagonist enzalutamide. Our data reveal novel mechanisms that underlie the regulation of PHF8 and KDM3A during NED and in CRPC, and support the candidacy of PHF8 as a therapeutic target in CRPC.

## INTRODUCTION

Prostate cancer (PCa) is the most common but second lethal malignancy in American men, with 180,890 new cases and 26,120 deaths estimated in 2016 [1]. Androgen-deprivation therapy (ADT), the most common treatment, initially results in PCa regression; however, two to three years after treatment castration-resistant prostate cancer (CRPC) develops [2]. The fact that CRPC patients have an overall survival rate of less than 3 years [3–5] highlights the importance of understanding the molecular etiology of CRPC and identifying novel therapeutic targets. The mechanisms underlying androgen-independent proliferation of CRPC cells include amplification of the androgen receptor (*AR*) gene, mutations in the *AR* gene, deregulation of AR co-regulators, ligand-independent activation of AR with or without elevated androgen synthesis, and AR-independent signaling [2, 6]. Evidence is accumulating that neuroendocrine-like (NE-like) cells, which express neuronal genes such as chromogranin A (*CHGA*), enolase 2 (*ENO2*) and synaptophysin (*SYP*), are present during PCa progression [7]. These cells can arise *via* neuroendocrine differentiation (NED), a process that can be induced by ADT and by the administration of therapeutic agents that target dividing cells, e.g. docetaxel [8]. NE-like cells do not proliferate and thus do not respond to the latter form of therapy. However, NED is reversible, such that a subset of NE-like cells can resume proliferation and contribute to cancer recurrence [8]. Thus, a deeper understanding of androgen-dependent and -independent mechanisms that promote NED, and consequently CRPC, is essential for identifying novel therapeutic targets for the treatment of CRPC.

The development of cancer has been shown to involve epigenetic mechanisms. Among these, histone methylation, which is dynamically regulated by methyltransferases and demethylases, is as important as other histone modifications in the epigenetic mechanisms of transcription regulation and genomic integrity [9]. Several histone demethylases and methyltransferase have been shown to promote the progression of PCa. For example, LSD1 (lysine-specific demethylase 1) demethylates H3K9me2/1 (di- and mono-methylated histone 3 lysine 9) to promote AR dependent transcription [10]. LSD1 also demethylates H3K4me2/1 to mediate the androgen-induced repression of AR gene itself in CRPC cells [11]. EZH2 (enhancer of zeste 2 polycomb repressive complex 2 subunit), an H3K27me3/2 methyltransferase, co-activates a subset of genes involved in AR-mediated gene transactivation specifically in CRPC cells [12]. Similarly, the H3K9me2/1 demethylase KDM3A/JmjD1A (lysine demethylase 3A) facilitates AR-mediated gene transactivation [13]. Moreover, KDM3A also serves as a transcription co-activator of HIF1α (hypoxia inducible factor 1 α subunit) and AR in the context of hypoxia [14]. The histone demethylase PHF8 (PhD finger protein 8) is a transcriptional co-activator by demethylating H4K20me1, H3K9me2/1 and H3K27me2 [15, 16]. PHF8 positively regulates the proliferation and migration of PCa cells [17, 18]. Although these epigenetic factors are known to contribute to PCa progression, how they are regulated during NED and the development of CRPC has not been systematically analyzed. Moreover, how PHF8 is regulated and whether it plays a role in NED and CRPC is not known.

In this study, we report a cluster of epigenetic factors following a unique expression pattern in the cell-based models of NED and CRPC. Mechanistically, we identified the c-MYC/*miR-22*/PHF8 axis and its connection with AR. We also show that PHF8 promotes proliferation and contributes to drug resistance in CRPC cells. These findings suggest that PHF8 might be a good candidate as a therapeutic target for treating CPRC.

## RESULTS

### Epigenetic factors that cluster based on expression in *in vitro* models of NED and CRPC follow a unique expression pattern

To identify novel epigenetic factors that are associated with NED and CRPC, particularly histone demethylases, we analyzed published gene expression profiles from cellular models of NED and CRPC. Given that androgen deprivation by treatment with charcoal-stripped FBS (CS-FBS) induces robust NED in LNCaP cells [19, 20], we defined the differentially regulated genes (DRGs) between LNCaP cells and LNCaP cells that had been treated with medium containing CS-FBS for 5 days (GSE51463) [20] as NED DRGs. To acquire CRPC DRGs, we incorporated LNCaP-Abl cells. LNCaP-Abl cells were generated from LNCaP cells by passaging them for over one year in CS-FBS medium, at which point they had acquired CRPC features [21]. Thus, we retrieved the DRGs between LNCaP and LNCaP-Abl cells (GSE39461) [12] using a standard ≥1.5 fold change and p<0.05 cutoff. Comparison between DRGs during NED (1061 upregulated and 692 downregulated gene entries) and DRGs in CRPC (7301 upregulated and 1651 downregulated gene entries) revealed six expression patterns of clustered genes: 1. Up-Up: upregulated during NED and in CRPC; 2. Up-Down: upregulated during NED but downregulated in CRPC; 3. UP in NED: upregulated during NED but restored in CRPC; 4. Down-Down: downregulated during NED and in CRPC cells; 5. Down-Up: downregulated during NED but upregulated in CRPC; 6. Down in NED: downregulated during NED but restored in CRPC (Table 1 and Supplementary File 1).

**Table 1:** Clustered epigenetic factors follow a unique expression pattern during *in vitro* NED and in CRPC

Patterns (No. of Genes)	Functional annotation* 1. Functions_categories, 2. Gene_Ontology, 3. Pathways	Representative genes or gene family
1. Up-Up (210)	1. Alternative splicing, transmembrane protein, membrane; 2. and 3. No significant enrichment	*ID1, VAV3, ATG3, MAPK10, UGT2B15, CD200, GALNT3, ADD2, AMIGO2, TGFBR3, 7 SLC* members
2. Up-Down (57)	1. Cell Adhesion, signal; 2. Homophilic cell adhesion, synaptogenesis, synaptic transmission, response to drug, transmission of nerve impulse, extracellular structure organization, neurological system process, cell projection organization; 3. No significant enrichment	*EPHA7, ADAM2, CD24, LAMB1, APLP1, CLSTN3, MAP1B, CDH3, PCDHB5/10/11/14/18*
3. Up during NED (454)	1. Signal, glycoprotein, alternative splicing, calcium, EGF-like domain, transmembrane, cell adhesion; 2. Cell adhesion, biological adhesion, cell motion, cell-cell adhesin. 3. Axon guidance	*CHGA, PCSK5, FN1, DDR1, FZD2/3 WNT2B, ITGA1, 3, MET, ITGB2/4/L1, GPR126/98/C5C, EPHB6, EFNB3, SLIT1, PCDHB15/16/6/8/GA8*
4. Down-Down (17)	No significant enrichment in all three categories	
5. Down-Up (232)	1. Cell cycle, mitosis, kinetochore, DNA replication, nucleus, phosphoprotein, ATP-binding, nucleotide binding, chromosomal protein, centromere, DNA damage and repair, Ubl conjugation, coiled coil, polymorphism, microtubule, motor protein, cytoplasm, cytoskeleton, meiosis, fanconi anemia, kinase, acetylation; 2. Cell cycle, M phase, DNA metabolic process, chromosome segregation, cellular response to stress, DNA strand elongation, centrosome cycle, DNA duplex unwinding; 3. Cell cycle, DNA replication, homologous recombination, pyrimidine metabolism, oocyte meiosis	*CDC6/25C/45/A3/A4/A5, CDT1, MCM2/3, CCNA2, CCNB2, CENPA/E/F/H/K/M/N/55, E2F1/2/3/8, NEK2, KLF11/15/22/23/2C/C1, HJURP, POLE2, BRCA1, BRCA2, AURKA, RAD54L, BLM, UBE2C, ASF1B, HIST1H1B, HIST1H4C, EZH2, UHRF1, SMC4, TOP2A, HMGB2*
6. Down during NED (249)	1. Secreted, signal, polymorphism, glycoprotein, disulfide bond, digestion, plasma. 2. No significant enrichment; 3. Neuroactive ligand-receptor interaction	*ADAM7, CD209, CD33, FGF21, HES6, MYC, VEGFA, KLK3, KLK4, NKX3-1*

* Select clusters with P-Value<0.001 from keywords in functional categories, BP_FAT in gene ontology, KEGG_Pathway in pathways are shown. The epigenetic factors in pattern 5 are underlined. Full gene lists for the six patterns are shown in supplementary file 1.

Analyses of functional categorization, gene ontology and pathways using DAVID (https://david.ncifcrf.gov) [22] revealed that genes involved in neuronal activities were significantly enriched in patterns 2 (Up-Down) and 3 (Up in NED), supporting a transient NE-like phenotype during NED. However, these genes were expressed at basal levels in LNCaP-Abl cells. The enrichment of cell cycle genes in pattern 5 (Down-Up) supports a reduction in cell proliferation during NED and an increase in CRPC cells. Many of the genes in this cluster were also upregulated in prostate tumors from the transgenic adenocarcinoma of the mouse prostate (TRAMP) mouse model, which develops spontaneous tumors in the prostate [23]. Importantly, this group also included epigenetic factors such as *EZH2*, *TOP2A*, *SMC4*, *UHRF1* and *HMGB2*. Interestingly, the expression of *EZH2*, *TOP2A* and *UHRF1* were also upregulated in human NEPC (neuroendocrine prostate cancer) [24], which share CRPC features, such as accelerated proliferation, androgen independency and poor prognosis [25]. Moreover, a study of patients tested after 22 weeks of ADT had revealed similar patterns, including downregulation of the cell cycle genes *TOP2A* and *UHRF1*, and upregulation of NED markers including *CHGA* and *ENO2* [26], supporting the physiological relevance of the *in vitro* models of NED and CRPC.

### PHF8 and KDM3A exhibit the down-up pattern in the *in vitro* models of NED and CRPC

Both histone demethylases PHF8 [17, 18] and KDM3A [13, 27] play oncogenic functions in PCa. Additional studies of KDM3A revealed that its demethylation of H3K9me2/1 facilitates the transcriptional activity of AR [13], and HIF1α in responding to hypoxia in prostate cancer cells [14]. Moreover, KDM3A contributes to the tumorigenesis of TRAMP-C cells, which were derived from tumors of TRAMP mice [28]. PHF8 [29] and KDM3A [30] play roles in the differentiation of neuronal precursors and endoderm, respectively. However, how these two histone demethylases are regulated and what roles they play during NED and in CRPC is not known. As our gene expression-based bioinformatics analysis did not reveal any histone demethylases, we hypothesized that PHF8 or KDM3A might be regulated post-transcriptionally and/or post-translationally during NED and in CRPC. We therefore tested this hypothesis using the *in vitro* models of NED and CRPC.

**Figure 1:**
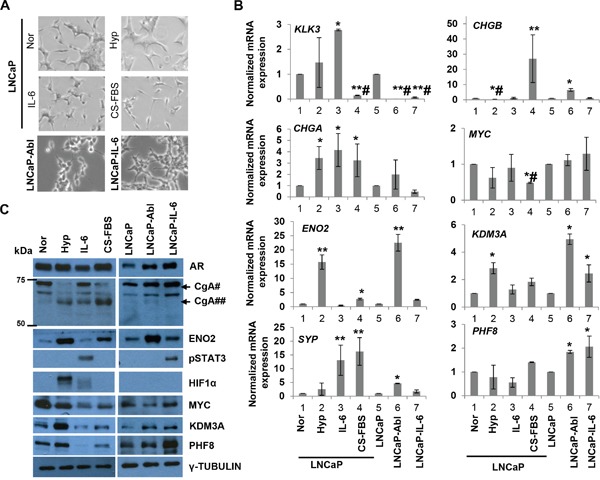
PHF8 and KDM3A are members of the Down-Up expression cluster in the *in vitro* models of NED and CRPC **A.** Phase-contrast images of LNCaP cells cultured under normal conditions (Nor) or following treatment with 1% O_2_ (Hyp), 20 ng/ml IL-6 (IL-6), or CS-FBS for 6 days, and LNCaP-Abl and LNCaP-IL-6 cells at steady state. **B.** RT-qPCR of indicated genes from cells cultured as in A. At least three independent experiments were performed, and standard deviation is indicated by bars. *: p<0.05; **: p<0.01; #: downregulation. **C.** Expression of the indicated proteins, as assessed by western blotting, in cells treated as in A. For CgA, the positions of molecular weight marks are shown. #: precursor CgA; ##: intermediate CgA.

In addition to CS-FBS treatment, hypoxic conditions [31] or interleukin-6 (IL-6) treatment [32] have also been reported to induce NED. To gain insight into the regulation of PHF8 and KDM3A during NED, we induced NED in LNCaP cells with CS-FBS medium, hypoxia (1% oxygen), or 20 ng/ml IL-6 for six days. Meanwhile, we acquired LNCaP-Abl and LNCaP-IL-6 cells as CRPC model cell lines. The LNCaP-IL-6 cell line had been generated from LNCaP cells by treatment with IL-6 for a long period of time (0.5 to 1 year) [33] and is able to undergo androgen-independent growth [34, 35]. Taken together, we expanded the number of the *in vitro* models of NED and CRPC by analyzing the effects of hypoxia and IL-6 on NED and utilizing both LNCaP-Abl and LNCaP-IL-6 cell lines as models of CRPC. We monitored the expression of the kallikrein related peptidase 3 (KLK3) mRNA as a control; In LNCaP cells, this mRNA was downregulated by CS-FBS treatment, upregulated by IL-6 treatment (Figure 1B), consistent with published data [36]. LNCaP-Abl and LNCaP-IL-6 cells express lower basal level of *KLK3* compared with that in LNCaP cells (Figure 1B), as previously reported [33, 37]. In contrast, the expression of *KLK3* did not increase in LNCaP cells cultured for six days under hypoxia, although an increase was reported in LNCaP cells cultured at less than 0.5% O_2_ for 4 to 18 hours [14, 38]. The expression of HIF1α and phosphorylated STAT3 (p-STAT3 Y705) was examined to validate the outcomes of treatment with hypoxia and IL-6, respectively (Figure 1C).

As expected, treatment with CS-FBS induced all features of NED, including upregulation of the *ENO2*, *CHGA*, *SYP* and *CHGB* mRNAs, as well as neuron-like morphological changes, e.g. increased neurite length (Figure 1A–1C and Supplementary Figure 1). Notably, ENO2 protein and a 65 kDa form of the CgA protein were also upregulated. Although the molecular weight for CgA in LNCaP cells has been reported to range from 65 kDa [20, 31] to 86 kDa [19], biochemical studies revealed that the CgA precursor is 75-80 kDa and that it is processed to intermediate fragments of 65 kDa and less [39]. Hence, it is likely that treatment with CS-FBS and hypoxia induce the processing of CgA in addition to its upregulation at the mRNA level. IL-6 induced the expression of *SYP*, *CHGA* mRNAs and CgA protein, as well as a partially neuron-like morphology (Figure 1A–1C and Supplementary Figure 1). Hypoxia induced the mRNA and protein expression of *ENO2* and *CHGA*, but not the mRNAs of *CHGB* or *SYP*. Hypoxia also induced a minor change in cell morphology (Figure 1A-1C and Supplementary Figure 1). These data indicate that IL-6 and hypoxia induce partial NED. On the other hand, LNCaP-Abl, but not LNCaP-IL-6 cells, expressed high levels of the *ENO2*, *SYP*, and *CHGB* mRNAs (Figure 1B) and ENO2 protein (Figure 1C) and exhibited a slight neuron-like morphology (Figure 1A). These data validated our cell-line based models of NED and CRPC, and indicated that some NE-like features are better reflected in LNCaP-Abl vs. LNCaP-IL-6 cells.

Next, we examined the expression of PHF8 and KDM3A in our models. We found that both proteins were upregulated by hypoxia but downregulated by treatment with IL-6 or CS-FBS (Figure 1C). At the mRNA level, *PHF8* was not significantly affected during the induction of NED, whereas *KDM3A* was significantly upregulated only by hypoxia (Figure 1B), consistent with a previous report [14]. Importantly, at both the mRNA and protein levels, PHF8 and KDM3A were significantly elevated in LNCaP-Abl and LNCaP-IL-6 cells (Figure 1B, 1C). Collectively, these results demonstrated that PHF8 and KDM3A follow the Down-Up expression pattern during NED inductions by androgen deprivation, IL-6 and in CRPC, supporting the involvement of post-transcriptional and/or post-translational regulatory mechanisms during NED. The elevated expression of both enzymes in CRPC cells implicates them in PCa progression.

### miR-22 mediates the regulation of PHF8 and KDM3A by IL-6

**Figure 2:**
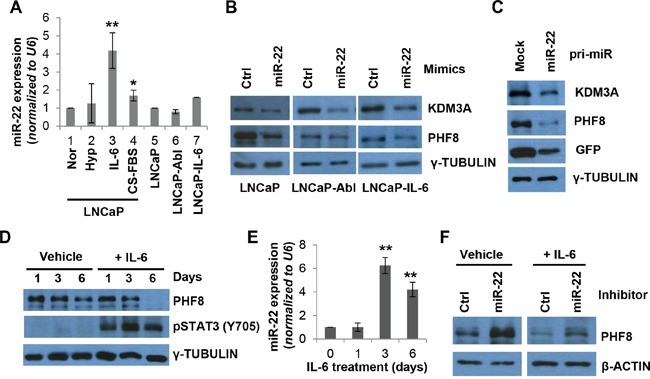
miR-22 mediates the regulation of PHF8 induced by IL-6 **A.** Expression of miR-22, as assessed by RT-qPCR, in LNCaP cells cultured under normal conditions (Nor) or following treatment with 1% O_2_ (Hyp), 20 ng/ml IL-6 (IL-6), or CS-FBS for 6 days, and in LNCaP-Abl and LNCaP-IL-6 cells. **B.** Expression of the indicated proteins, as assessed by western blotting, in LNCaP, LNCaP-Abl, and LNCaP-IL-6 cell lines following transfection with 20 nM control (ctrl) or miR-22 mimics for 48 hours. **C.** Expression of the indicated proteins, as assessed by western blotting, in LNCaP cells stably overexpressing the GFP (mock) only or GFP-miR-22 construct. **D.** Expression of the indicated proteins, as assessed by western blotting, in LNCaP cells treated with medium containing 0.1% BSA (Vehicle) or medium containing 20 ng/ml IL-6 (+IL-6), for the indicated number of days. **E**. Expression of miR-22, as assessed by RT-qPCR, in cells treated with IL-6 as in D. **F.** Expression of the indicated proteins, as assessed by western blotting, in LNCaP cells transfected with 50 nM control (Ctrl) or miR-22 inhibitor at 72 hours following treatment with vehicle or IL-6 (+IL-6). S.D. in all experiments was obtained from at least three independent experiments. *: p<0.05; **: p<0.01.

Given that PHF8 and KDM3A are not transcriptionally regulated by short treatment with either IL-6 or CS-FBS, we sought to identify post-transcriptional mechanisms that might be involved. One reason for this is that the regulator(s) themselves may play key roles mediating the dynamic changes of gene networks both during NED and in CRPC. Analysis using TargetScan [40] revealed that the target seed sequences of several conserved microRNAs (miR-31, -182, -9, -22 and members of the let-7 family) are present in the *PHF8* 3′ UTR (Supplementary Figure 2A and 2B). Among these microRNAs, only miR-22 was reported to be upregulated when LNCaP cells were treated with either CS-FBS or the AR antagonist bicalutamide [41]. Notably, miR-22 is also targeted and upregulated by AR [42], suggesting complexed regulatory mechanisms of miR-22 expression. In our NED-CRPC cell system, the expression of miR-22 was upregulated by treatment with CS-FBS, consistent with the previous study [41]. Surprisingly, this was also the case for treatment with IL-6 (Figure 2A). However, miR-22 was not significantly upregulated in either LNCaP-Abl or LNCaP-IL-6 cells. Since, miR-22 was reported to target and regulate KDM3A in Ewing sarcoma [43], these findings suggest that miR-22 may regulate both PHF8 and KDM3A in LNCaP cells, at steady state as well as in the presence of CS-FBS or IL-6.

To determine if miR-22 directly targets the *PHF8* 3′ UTR and regulates its expression, we retrieved data from Ago2 CLIP-seq studies using starBase v2.0 [44, 45]. Indeed, miR-22 binding sites within the *PHF8* 3′ UTR were pulled down by Ago2, implicating miR-22 in directly targeting *PHF8* (Supplementary Figure 2A). In LNCaP cell lines stably expressing either pLenti-GFP-empty or pLenti-GFP-*PHF8* 3′ UTR, transient transfection of miR-22 mimics, but not control mimics, significantly downregulated GFP-*PHF8* 3′ UTR. Such transfection did not affect the expression of GFP-empty (Supplementary Figure 2C). Importantly, the GFP-*PHF8* 3′ UTR showed consistent lower expression compared with the GFP-empty vector, implicating that *PHF8* 3′ UTR is subject to repression. Transient transfection of miR-22 mimics also downregulated the expression of PHF8 and KDM3A in LNCaP, LNCaP-Abl and LNCaP-IL-6 cells (Figure 2B). Moreover, stable overexpression of *pri-miR-22* in LNCaP cells led to a reduction of both demethylases at the protein level (Figure 2C and Supplementary Figure 2D), supporting our hypothesis that miR-22 regulates PHF8 and KDM3A in PCa cells.

Since miR-22 is more profoundly upregulated by IL-6 than by the treatment with CS-FBS, we asked if it is involved in the regulation of PHF8 in this context. A close examination of the expression of PHF8 and miR-22 revealed that the miR-22 elevation peaked at 72 hours, preceding the downregulation of PHF8 at the protein, but not mRNA, level (Figure 2D, 2E and Supplementary Figure 3). Indeed, transient transfection of the cells with miR-22 inhibitors 72 hours after initiation of IL-6 treatment partially restored the levels of PHF8 protein (Figure 2F), indicating that miR-22 is involved in IL-6 induced downregulation of PHF8. As levels of the PHF8 mRNA did not change during IL-6 treatment, miR-22 likely inhibits the translation of PHF8. Taken together, our data revealed that IL-6 upregulates miR-22, which mediates the downregulation of PHF8 in this context. Such a mechanism may also contribute to the regulation of PHF8 and/or KDM3A in response to CS-FBS treatment given the elevated expression of miR-22 during NED induction by both IL-6 and CS-FBS. We next asked if miR-22 plays a role in NED. Transient transfection of miR-22 mimics in LNCaP cells slightly increased the length of neurites (Supplementary Figure 4A and 4B), increased the intermediate CgA protein and the mRNA level of *CHGB* (Supplementary Figure 4C). However, the *CHGA* mRNA is downregulated (Supplementary Figure 4D). These data implicate that transient miR-22 overexpression contributes to NED induction, but, does not induce full NED.

### MYC sustains the expression of PHF8 and KDM3A by repressing miR-22

**Figure 3:**
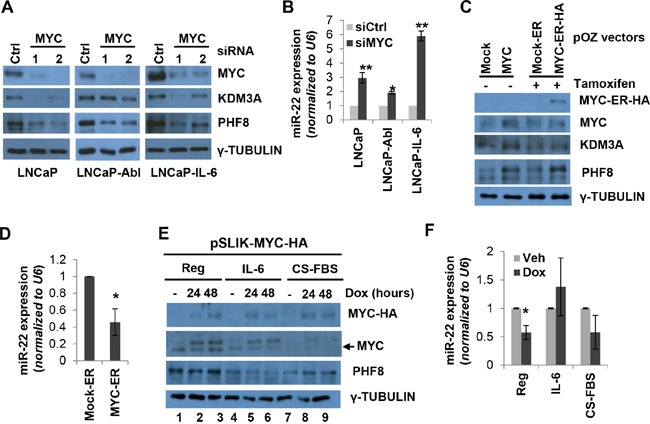
MYC sustains the expression of PHF8 and KDM3A by repressing miR-22 **A.** Expression of the indicated proteins, as assessed by western blotting, in LNCaP, LNCaP-Abl and LNCaP-IL-6 cells treated with 20 nM scrambled (Ctrl) or MYC siRNA for 60 Hours. **B.** Expression of miR-22, as assessed by RT-qPCR, in cells treated as in A. **C.** Expression of the indicated proteins, as assessed by western blotting, in LNCaP cells over-expressing a constitutively active MYC construct and a tamoxifen-inducible mock construct (mock-ER) or MYC-ER-HA. 1 μM tamoxifen was added to the medium for 48 hours. **D.** Expression of miR-22 in LNCaP cells overexpressing mock-ER or MYC-ER-HA as in C. **E.** Expression of the indicated proteins, as assessed by western blotting, in LNCaP cells over-expressing a doxycycline-inducible MYC-HA construct and treated with medium containing vehicle (Reg), 20 ng/ml IL-6 (IL-6) or CS-FBS (CS-FBS) for 6 days. Doxycycline (dox) was used at 0.5 μg/ml for the indicated amount of time. **F.** Expression of miR-22 in LNCaP cells treated as in E as assessed by RT-qPCR. The expression of miR-22 was normalized to the vehicle treatment (no doxycycline). All experiments were performed at least three times independently, and standard deviation is indicated by bars. *: p<0.05; **: p<0.01.

c-MYC (hereafter referred to as MYC)-mediated repression of miR-22 has been reported in several cell types [46, 47], yet it is unclear whether this occurs in PCa cells. Since the expression of MYC protein is similar to that of PHF8 and KDM3A following treatment of LNCaP cells with IL-6 and CS-FBS (Figure 1B and 1C), we hypothesized that MYC sustains the expression of PHF8 and KDM3A by repressing miR-22, both during NED and in CRPC. siRNA-mediated MYC knockdown in LNCaP, LNCaP-Abl and LNCaP-IL-6 cells led to reduced expression of the PHF8 and KDM3A proteins, and to increased expression of miR-22 (Figure 3A and 3B). Notably, the effects of MYC knockdown on the reduction of PHF8 and KDM3A protein levels *via* miR-22 were greater in LNCaP and LNCaP-IL-6 cells than in LNCaP-Abl cells, implying that MYC plays distinct roles in these contexts. In contrast, stable or tamoxifen-induced MYC overexpression in LNCaP cells upregulated the expression of PHF8 and KDM3A protein and downregulated that of miR-22 (Figure 3C and 3D).

To test if MYC mediates the regulation of PHF8 in response to IL-6 and CS-FBS, we established a LNCaP cell line stably expressing doxycycline-inducible MYC. The induction of MYC rescued the PHF8 downregulation that normally occurs in the context of treatment with CS-FBS (Figure 3E, compare lane 7 with 8 and 9), and is accompanied by marginal downregulation of miR-22 (Figure 3F). Interestingly, in the context of IL-6 treatment, MYC induction failed to repress miR-22 (Figure 3F) and to restore PHF8 protein levels (Figure 3E, compare lane 4 with 5 and 6). Given that IL-6 enhances AR activity [36] and AR contributes to activation of miR-22 [42], it is likely that IL-6-mediated miR-22 induction is more profoundly influenced by AR activation than by de-repression of downregulated MYC.

### MYC and PHF8 exhibit similar responses to exogenous IL-6 in cells with partial or full autocrine IL-6 capacity

**Figure 4:**
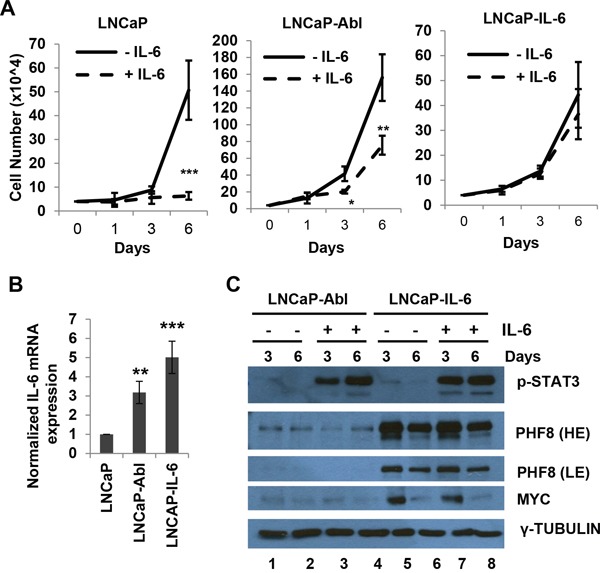
MYC and PHF8 cluster in responding to exogenous IL-6 in cells that are capable of either partial or full autocrine IL-6 signaling **A.** Cell number in cultures of LNCaP, LNCaP-Abl and LNCaP-IL-6 cells, obtained at the indicated time points over a 6-day period during treatment with regular medium (-IL-6) or medium containing 20 ng/ml IL-6 (+IL-6). **B.** Expression of *IL-6*, as assessed by RT-qPCR, in LNCaP, LNCaP-Abl and LNCaP-IL-6 cells. **C.** Expression of the indicated proteins in LNCaP-Abl and LNCaP-IL-6 cells treated with medium containing vehicle (-) or 20 ng/ml IL-6 (+) for the indicated number of days. HE: High Exposure; LE: Low Exposure. *: p<0.05; **: p<0.01; ***: p<0.005.

To further investigate the link between MYC and PHF8, we tested how the two proteins respond to IL-6. We used LNCaP and LNCaP-IL-6 cells, which are capable of paracrine and autocrine IL-6 signaling, respectively. In addition, we also included LNCaP-Abl cells, as they represent a model of CRPC, but with unclear secretion status of IL-6. IL-6 treatment inhibited the proliferation of LNCaP cells, but not that of LNCaP-IL-6 cells (Figure 4A), consistent with a previous report [33]. The same treatment only partially inhibited the growth of LNCaP-Abl cells (Figure 4A). RT-PCR showed that LNCaP-IL-6 and LNCaP-Abl cells expressed high and moderate levels of *IL-6* mRNA, respectively. Such expression was nearly absent in LNCaP cells (Figure 4B). These results indicate that LNCaP-Abl cells are partially competent for autocrine IL-6 signaling, a finding that may explain the partial resistance of these cells to the growth inhibitory function of exogenous IL-6. Although exogenous IL-6 induced phosphorylation of STAT3, it did not dramatically affect the expression of MYC and PHF8 proteins in LNCaP-Abl and LNCaP-IL-6 cells (Figure 4C, compare lanes 2 and 4, lanes 6 and 8) than that in LNCaP cells (Figure 1C). Notably, both PHF8 and MYC were slightly downregulated in LNCaP-IL6 cells after 6 days of treatment regardless of whether the medium contained IL-6. It is possible that the downregulation of both MYC and PHF8 reflects the point at which the cells reached their proliferation plateau, a cellular phase described previously to alter proliferative gene expression [48]. In sum, the sustained PHF8 and MYC protein levels in both LNCaP-IL-6 and LNCaP-Abl cells tighten the link between MYC and PHF8, and indicates that these two proteins may play key roles in resistance to growth inhibition induced by exogenous IL-6.

### AR is upstream of the MYC/miR-22/PHF8 axis in CRPC cells

**Figure 5:**
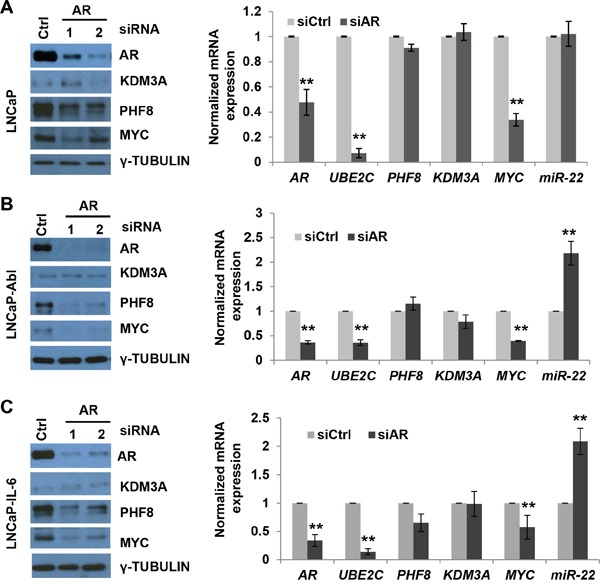
AR is upstream of the MYC/miR-22/PHF8 axis in CRPC cells **A-C** Expression of the indicated proteins, as assessed by western blotting, and of the indicated mRNAs, as assessed by RT-qPCR, in LNCaP, LNCaP-Abl and LNCaP-IL-6 cells transfected with 20 nM of a scrambled siRNA (Ctrl) or an AR siRNA for 60 Hours. *: p<0.05; **: p<0.01.

Both AR-dependent and -independent pathways contribute to CRPC development [6]. Thus, an understanding of the relationship between AR and the MYC/miR-22/PHF8 axis is critical to evaluating the potential therapeutic significance of inhibiting PHF8 in combination with the application of AR antagonists. The efficiency of AR knockdown by siRNAs was verified for all three cell lines (Figure 5), and the impact on target gene expression was validated by the downregulation of UBE2C (Figure 5), whose relevance to AR signaling has previously been demonstrated [37]. In all three cell lines, AR knockdown downregulated PHF8 at the protein level without affecting expression of the mRNA. In contrast, KDM3A was unaffected at both the protein and mRNA levels (Figure 5), and MYC was downregulated at both the protein and mRNA levels (Figure 5), consistent with a previous report [49]. Expression of miR-22, in contrast, was de-repressed only in LNCaP-Abl and LNCaP-IL-6 cells (compare Figure 5A to 5B and 5C). Given the fact that miR-22 is subject to activation by AR [42] and to repression by MYC (Figure 3B), the upregulation of miR-22 in LNCaP-Abl and LNCaP-IL-6 cells suggests that the repression by MYC is dominant. In LNCaP cells, the unaffected miR-22 expression suggests that AR and MYC play regulatory roles in activating and repressing miR-22 expression, respectively. In the case of LNCaP cells, AR knockdown may regulate PHF8 *via* alternative mechanisms. Additionally, at steady state miR-22 expression is not as high in LNCaP-Abl and LNCaP-IL-6 cells as in LNCaP cells (Figure 2A), despite the fact that AR levels are high in these CRPC cell lines (Figure 1C). Thus, activation by AR appears to supersede repression of miR-22 by MYC. These complex regulatory mechanisms are illustrated in Figure 8. Taken together, these data demonstrate that PHF8 is downstream of AR, and that it is regulated by the MYC/miR-22 axis in CRPC cells.

### PHF8 is co-expressed with AR in clinical PCa samples and early prostate tumors from TRAMP mice

**Figure 6:**
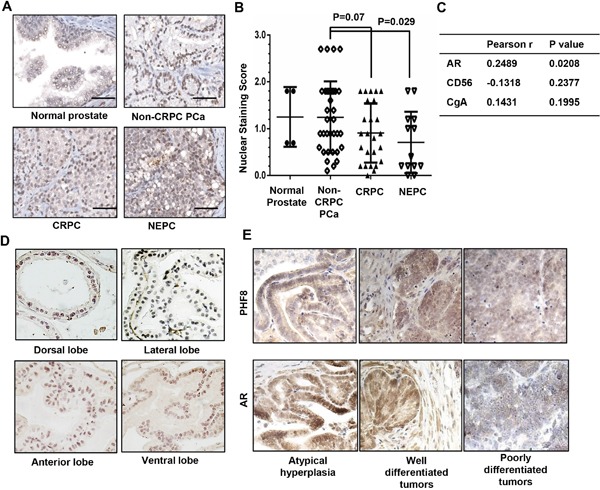
PHF8 is co-expressed with AR in clinical PCa samples, in normal mouse prostate, and in prostate tumors from TRAMP mice **A.** Representative images (40X) of PHF8 IHC staining in human PCa tissue array. **B.** Distribution of scores for PHF8 expression for 4 cores from normal prostate tissue, 30 cores from non-CRPC PCa, 25 cores from CRPC, and 13 cores from NEPC. **C.** Pearson correlation of expression of PHF8 with that of AR, CD56 and CgA. **D** and **E.** Representative images (40X) of PHF8 IHC staining in normal prostate tissue (N=5) and tumors from TRAMP mice aged 3 to 5 months (N=7).

The elevated expression of PHF8 in CRPC cells and the post-transcriptional regulatory mechanisms whereby AR regulates PHF8 expression prompted us to test clinical CRPC samples for correlation of the expression of the two proteins. We carried out immunohistochemical (IHC) staining of PHF8 on a tissue array containing 2 cases of normal prostate tissues, 20 cases of non-CRPC PCa samples, 14 cases of CRPC samples and 6 cases of NEPC samples. NEPC does not express AR, but shares epigenetic factors, e.g. *EZH2*, *TOP2A* and *UHRF1* with CRPC [24], thus, providing a platform to examine if PHF8 is clustered with these epigenetic factors. We confirmed that PHF8 stained the nuclei, and scored the staining intensity by the SL801 autoloader and Leica SCN400 scanning system (Figure 6A). Our statistical analysis shows that PHF8 expression does not differ significantly (p=0.07) between CRPC and non-CRPC PCa samples (Figure 6B). The comparable expression of PHF8 in CRPC and non-CRPC PCa suggest that PHF8 protein is upregulated in CRPC cells, as it was reported that PHF8 protein is upregulated in 80% of 332 PCa samples [17]. Moreover, the 6 NEPC cases show non-detectable to high levels of PHF8 expression (Figure 6A, 6B). Although the statistical analysis shows a significant difference (p=0.029) in PHF8 expression between NEPC and non-CRPC PCa, due to the low number of cases of NEPC (N=6) further IHC studies on such samples will be needed before a conclusion can be drawn about PHF8 expression in NEPC. Importantly, further analysis showed that in all PCa samples examined the expression of PHF8 is significantly correlated with that of AR but not that of NED markers CD56 or CgA (Figure 6C).

Next, we investigated PHF8 expression in normal prostate and prostate tumors from the TRAMP mouse, an animal model that has been widely used for PCa studies [50]. PHF8 was detectable in all lobes of normal mouse prostates (N=5), with strong staining in the epithelia of the anterior and ventral lobes (Figure 6D). In prostate tumors from these mice (N=7), PHF8 expression was detectable at the stage of atypical hyperplasia and in well differentiated tumors, but was low or non-detectable in the poorly differentiated tumors (Figure 6E and Supplementary Figure 5). Importantly, the expression of PHF8 followed the same pattern as that of AR (Figure 6E). In sum, PHF8 and AR were confirmed to be co-expressed in human PCa samples, mouse prostate, and mouse prostate tumors, supporting our cell line-based findings that AR positively regulates PHF8.

### PHF8 promotes cell proliferation by regulating cell cycle genes and sensitizes LNCaP-Abl cells to enzalutamide treatment

**Figure 7:**
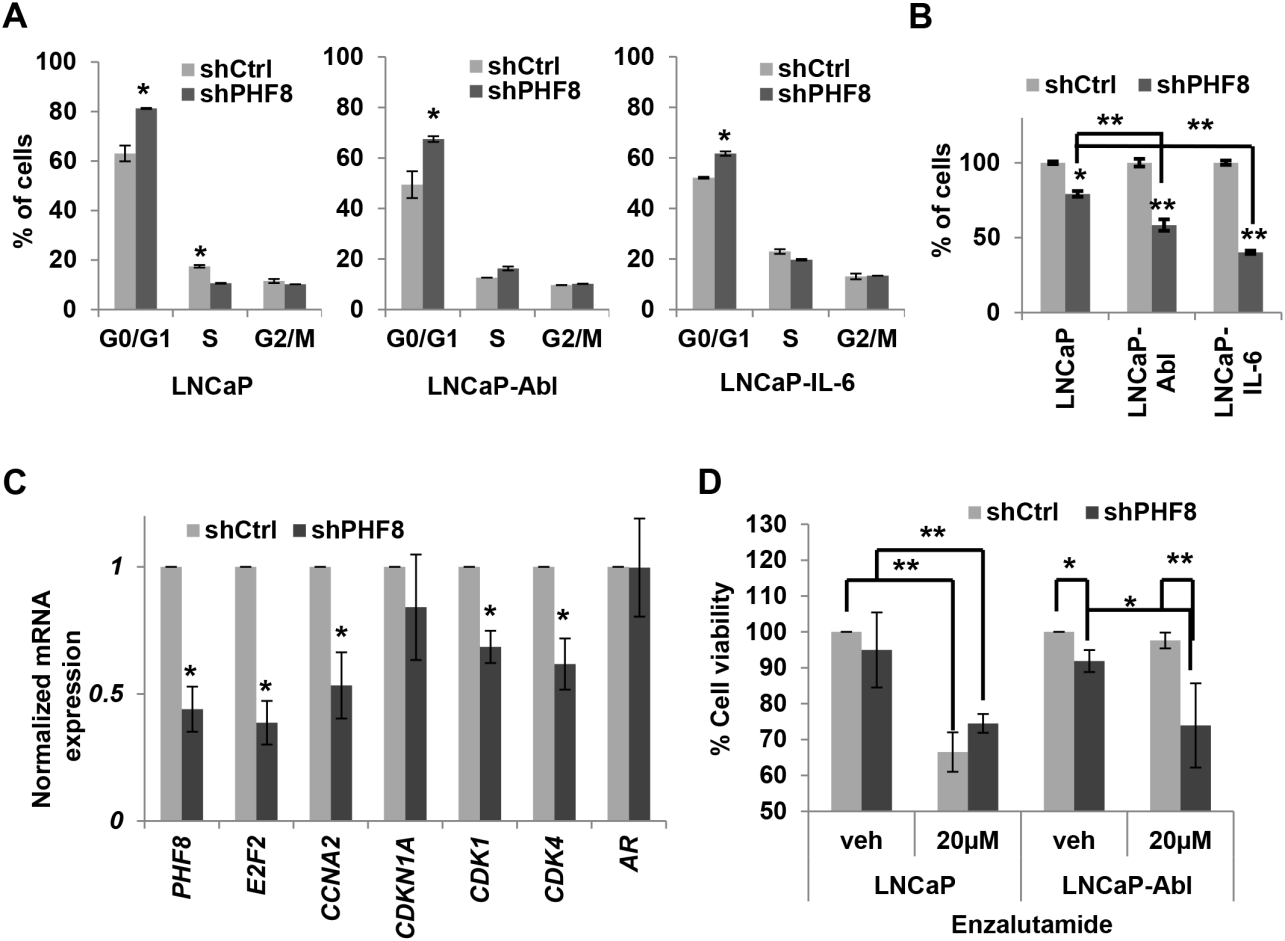
PHF8 promotes proliferation by regulating cell cycle genes and sensitizes LNCaP-Abl cells to enzalutamide treatment **A.** Cell cycle distributions of LNCaP, LNCaP-Abl and LNCaP-IL6 cells stably expressing doxycycline-inducible scrambled shRNA (Ctrl) or a PHF8 shRNA. **B.** Percentage of cells in indicated cultures, as described in A. **C.** Expression of the indicated genes, as assessed by RT-qPCR, in LNCaP-Abl cells treated as in A. **D.** Cell viability, as determined by MTT assay, in LNCaP and LNCaP-Abl cells in which PHF8 knockdown was induced for 48 hours before DMSO (veh: vehicle) or 20 μM enzalutamide was applied for another 48 hours. All experiments were performed at least three times independently, and standard deviation is shown by bars. *: p<0.05; **: p<0.01.

Based on the connection between PHF8 and AR, we sought to determine if PHF8 plays a role regulating the proliferation of CRPC cells. Two siRNAs and one doxycycline-inducible shRNA against PHF8 were used; they all knocked down PHF8 efficiently (Supplementary Figure 6A). Both PHF8-siRNA1 and the shRNA caused significant accumulation of cells in G0/G1 in LNCaP, LNCaP-Abl and LNCaP-IL-6 cells (Figure 7A and Supplementary Figure 6B). Given that PHF8 knockdown had been reported to affect cell cycle progression in LNCaP cells [17] and we had previously found that this shRNA abolished target-gene binding by PHF8 [15], we used it for our further studies. Knockdown of PHF8 significantly reduced proliferation in all three cell lines, but had more profound effects on LNCaP-IL-6 and LNCaP-Abl cells than on LNCaP cells (Figure 7B). Focusing on LNCaP-Abl cells, PHF8 knockdown significantly downregulated the expression of several cell cycle genes (Figure 7C). Furthermore, PHF8 knockdown reduced the viability of LNCaP-Abl cells at steady state, and sensitized them to cell viability inhibition of enzalutamide (Figure 7D). In contrast, PHF8 knockdown did not dramatically affect the viability of LNCaP cells, either at steady state or in the context of enzalutamide treatment (Figure 7D). These results further support the profound requirement for PHF8 in the survival of LNCaP-Abl cells.

## DISCUSSION

Identifying novel epigenetic factors that play important roles during PCa progression is critical for the development of new drug targets for the treatment of CRPC. Taking advantage of published gene expression data from LNCaP cells that have been deprived of androgen for short (NED induction) and long (CRPC features) periods and that have been used to model the progression from ADT treatment to CRPC, we identified six patterns of clustered genes. We discovered that the epigenetic factors *EZH2*, *UHRF1*, *SMC4*, *TOP2A* and *HMGB2* are clustered and that their expression is downregulated during NED but upregulated in CRPC (Down-Up pattern). Importantly, EZH2 [12] and UHRF1 [51] have been shown to contribute to PCa progression, providing validation of our expression analysis. Our findings added PHF8 and KDM3A to the cluster of the epigenetic factors in the Down-Up pattern.

From a mechanistic standpoint, our study revealed that MYC regulates PHF8 and KDM3A through miR-22 in both androgen-dependent and -independent LNCaP cells, as well as during the induction of NED by treatment with IL-6 and CS-FBS. The oncogenic functions of MYC in PCa have been well documented; its amplification is often associated with PCa progression and poor clinical outcome [52, 53], and it plays critical roles in androgen-independent PCa progression [49, 54]. Notably, MYC gain-of-function mutations can enhance the proliferation of cancer cells, the metastasis of cancers, and instability of the genome [55]. Our study positions MYC upstream of PHF8, a demethylase that regulates H4K20me1, which is important for kinetochore assembly and key to maintaining genome stability [56]. Whether upregulated PHF8 mediates MYC function in driving genome instability in the context of PCa is worth further investigation.

miR-22 has been reported to be downregulated in PCa [42, 57], and the use of miR-22 mimics inhibits the migration of both LNCaP and PC3 cells [42]. In this regard, it is notable that miR-22 targets and regulates *IPO7* [57] and *LAMC1* [42], both of which are overexpressed and have oncogenic functions in PCa. Moreover, miR-22 also targets MYCBP1 to downregulate MYC function [58]. This evidence supports the tumor repressive function of miR-22. In contrast, miR-22 has been reported to be upregulated in PCa, where it downregulates expression of the tumor suppressor gene pTEN [59]. Our analysis shows that treatment with IL-6 or CS-FBS leads to upregulation of miR-22, but that this activation is lost when cells take on an IL-6-producing, androgen-independent phenotype. These findings indicate that the expression of miR-22 is dynamically regulated. Given that both the activation of MYC and loss of the PTEN tumor suppressor are frequently observed in PCa, and when these abnormalities are combined in mouse models they drive genome instability and metastasis of PCa [60], it will be important to carefully evaluate the functions of miR-22 in PCa in the context of its upstream regulatory signals and downstream target genes. In the cases of PCa in which MYC is amplified, the downregulation of miR-22 may cause an increase in the expression of PHF8 and KDM3A. Moreover, *PHF8* 3′UTR may contain consensus target seed sequences for miRs other than miR-22 (miR-31, miR-182, miR-9 and let-7). Whether these microRNAs are regulated by MYC or AR during NED and in CRPC cells, and if they regulate PHF8 remain open questions.

The high level of induction of miR-22 expression by IL-6, which reflects the dominant transactivation of miR-22 expression by AR, may play important additive roles in the induction of NED. For example, if PHF8 and KDM3A have pro-proliferative functions their repression will facilitate cell cycle arrest, which could be a pre-condition for induction of NED or be simultaneously required. Our findings that miR-22 mimics slightly increase the length of neurite, intermediate CgA and the mRNA level of CHGB suggest that miR-22 plays an additive role, with other factors dominant in NED induction.

Both AR and IL-6 signaling pathways play critical roles in the initiation and progression of PCa [6, 61]. In contrast, the expression of AR and its transactivity are often inversely correlated with NED and NE-like cells, and AR represses NED [62, 63]. Although several studies have demonstrated that NED can be induced by IL-6 [32, 64, 65], the elevated AR activity induced by IL-6 may counteract the induction of NED. This phenomenon was observed in our study in which IL-6 treatment induced the expression of two out of four NED markers and resulted in a partial change to a NE-like cell morphology. Although we propose that an AR/MYC/miR-22/PHF8 regulatory axis exists, we acknowledge that this axis is likely context dependent. In the case of brief treatment with IL-6, for example, AR activity is increased but MYC is downregulated, indicating that MYC expression becomes uncoupled from AR in this situation. Notably, the link between AR and MYC is restored when LNCaP cells gain IL-6 autocrine function (LNCaP-IL-6 cells).

**Figure 8:**
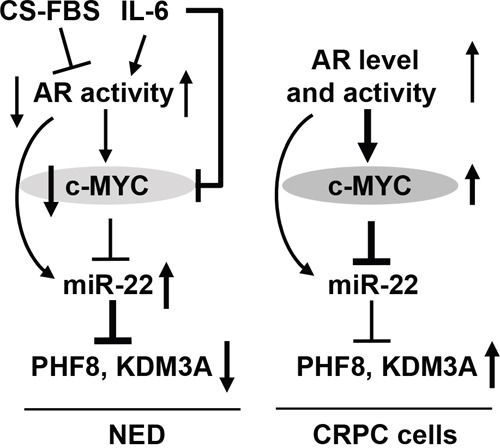
Schematic illustration of the mechanisms underlying the regulation of PHF8 and KDM3A by AR, MYC and miR-22 DuringNED (left), androgen deprivation reduces AR activity and MYC expression, miR-22 is derepressed from the downregulated MYC and consequently, PHF8 and KDM3A are downregulated by the elevated miR-22. Short IL-6 treatment downregulates MYC, although, it increases AR activity. The upregulation of miR-22 can be caused by the elevated AR activity and the derepression from the downregulated MYC. In CRPC cells (right), miR-22 is repressed by MYC, despite of elevated AR activity. The basal expression level of miR-22 partially contributes to the restored expression of PHF8 and KDM3A in CRPC cells.

In sum, we have discovered a novel regulatory axis that involves AR, MYC, miR-22, PHF8 and KDM3A and that functions during NED and in CRPC cells, as illustrated in Figure 8. The high expression and proliferative function of PHF8 in CRPC cells support its candidacy as a therapeutic target for patients with advanced PCa.

## MATERIALS AND METHODS

### Cell culture, cell cycle profiling, cell proliferation and cell viability assays

Low passage-number (<10 passages) LNCaP, Phoenix A, and 293ET cells were obtained from ATCC. LNCaP-Abl (passage 75) and LNCaP-IL-6 (passage 46) cells were kind gifts from Dr. Zoran Culig (University of Innsbruck, Austria). LNCaP and LNCaP-IL6 were maintained in RPMI 1640 medium, whereas PhoenixA and 293ET cells were maintained in high-glucose DMEM (Life Technologies). Both media contained, 10% Fetal Bovine Serum (FBS), 100 units/ml Penicillin, 100 μg/ml Streptomycin, 1mM Sodium Pyruvate and 15 μg/ml Plasmocin™ prophylaxis (InvivoGen). LNCaP-Abl cells were cultured in RPMI medium supplemented with 10% CS-FBS. A humidified hypoxia chamber (Coy Labs) calibrated to 1% O_2_ using a 95%N_2_/5% CO_2_ gas mixture was used for hypoxia treatment. IL-6 (PeproTech) at 20 ng/ml was used to treat the cells, with the medium changed every 48 hours. Cells seeded at a density of 142 cells/mm^2^ and 38 cells/mm^2^ were used for <96 hr and 6 day experiments, respectively.

For cell cycle profiling, cells were trypsinized, washed with ice-cold PBS, fixed with 80% ethanol/PBS on ice for 30 minutes and stained for 30 minutes at 37°C with 50 μg/ml propidium iodide containing 250 μg/ml RNAse (Sigma). Cells were filtered and analyzed on an LSR-UV Flow Cytometer (BD Biosciences). Cells stably expressing florescent proteins were prepared as above and sorted on a FACS Aria machine (BD Biosciences). Cell cycle profiles were analyzed using FlowJo 9™ software and the Dean-Jet fitting algorithm.

Enzalutamide (Selleckchem) was applied at 20 μM to target cell lines for 48 hours prior to MTT assays. Briefly, the MTT solution was added to each well at a final concentration of 5 μg/ml, followed by 4-hour incubation and dye dissolution in DMSO. The absorbance was read at 490 nm on a microplate reader (Molecular Devices). Cell proliferation assay was performed by cell counting using a hemacytometer (Hausser Scientific Inc).

### Plasmids and stable cell lines

MYC2 (NM_002467) and MYC-ER from a pBabe-MYC-ER plasmid (Addgene plasmid#19128) [66] were cloned into the XhoI and NotI sites of the pOZ retroviral vector, and into the SpeI and XhoI sites of the pEN_TTmcs vector (Addgene plasmid# 25755) [67]. The pEN_TTmcs-empty and -MYC-HA vectors were recombined into pSLIK-hygro vector using Gateway Technology according to the manufacturer's instructions (Invitrogen). Pri-*miR-22* (locus NR_028502) was cloned into the BglII and XhoI sites of pMSCV-PIG. Control GFP and PHF8 shRNAs [15] were cloned into the AgeI and EcoRI sites of a TetON-pLKO-puro vector, which was a gift from Dmitri Wiederschain (Addgene plasmid # 21915) [68]. *PHF8* 3′ UTR (2485 bp) was cloned into the SalI and EcoRI sites of the pLenti-GFP-puro vector.

Retrovirus packing of the pOZ, pMSCV-PIG vectors, infection, and stable selections were performed as described previously [15]. Additionally, pMSCV-PIG-pri-*miR-22* LNCaP cells were FACS sorted to enrich for GFP-positive clones. Lentiviruses containing the Tet-pLKO-puro, pSLIK-hygro, or pLenti-GFP-puro vector were packaged using Lenti-X 293T cells (Clontech) and 1 μg/ml puromycin or 50 μg/ml hygromycin (Life technologies) were used for stable selection. 0.5 μg/ml doxycycline and1 μM tamoxifen (Sigma) were applied to induce target transgene expression. Doxycycline and tamoxifen were refreshed every 48 or 24 hours, respectively.

### Transfections, western blotting, antibodies and RT-qPCR

miR-22 mimics (GenePharma) or inhibitors (Integrated DNA Technologies) were used at a final concentration of 20 nM or 50 nM, respectively. siRNA duplexes (OriGene and Sigma) targeting the human AR (siRNA1 GCCUUUAAAUCUGUGAUGAUCCUCA, siRNA2 GGACUUUCCGGAAAUGAUGGCAGAG), PHF8 (siRNA1 AGCAAAGAAGGTAGACAAGGCTA GG, siRNA2 GGAGGACTATACAACAGATGAGGAC) and MYC (siRNA1 CCGAGGAGAAUGUCAAG AGGCGAAC, siRNA2 CGUCCAAGCAGAGGAGCAA) were used at a final concentration of 20 nM for 60 hours. All transfections of RNA duplexes were performed using the Lipofectamine RNAiMAX transfection reagent (Life Technologies).

For western blotting, cells were lysed in 2% SDS or RIPA buffer (50mM Tris-HCL pH 7.4, 150mM NaCl, 0.1% SDS, 0.5% Sodium Deoxycholate, 1% Triton-X) supplemented with protease inhibitor (Sigma), 10mM NaF, 1mM DTT, and 0.5mM NaVO_3_ before being briefly sonicated using a QSonica® Q700 sonicator (Qsonica), and protein levels were normalized using Bradford Protein Assay (BioRad). 15-30 μg of total protein was loaded onto gels, blotted and probed with the following primary antibodies: KDM3A (A301-539A, Bethyl Labs), PHF8 in house rabbit-anti-PHF8 [15] for most of western blotting, Chromogranin A/CgA (MS-324-P0), γ-TUBULIN (MA1-850) from Thermo Fisher; AR (SC-816), c-MYC (SC-40), pSTAT3 (SC-7993), GFP (SC-9996) from Santa Cruz; HIF1α (610958 DB Bioscience), β-ACTIN (Ab8287, Abcam), ENO2 (M0873, DAKO), and HA (MMS-101P Covance). Western blot intensities were quantified using Adobe Photoshop.

For RT-qPCR, total RNA was first isolated using TRIZOL (Ambion) following the manufacturer's protocol. 1 μg RNA was reverse transcribed using MMLV reverse transcriptase according to manufacturer's protocol (Promega Inc) and the cDNA was diluted 1:3 for NED marker genes, or 1:10 for most other genes. MicroRNA cDNA was synthesized as previously described [69] and diluted 1:5 in the case of the target microRNA and 1:100 in the case of the *U6* loading control. Diluted cDNA was subjected to quantitative PCR using the iScript™ Advanced SBYR Green dye and a CFX96 instrument (BioRad). Data were analyzed using the ΔΔC_t_ method. See Supplementary Table 1 for list of RT-qPCR primers used.

### Mice, histology and immunohistochemistry

Animal studies were performed in accordance with practices and standards outlined by the University of Iowa Animal Facility-Office of Animal Resources. TRAMP-FVB mice (stock # 008215) were purchased from Jackson Laboratory and backcrossed onto the FVB-NJ strain (stock # 001800) for at least 3 generations before analysis. Genotyping was performed by following the Jackson Laboratory protocol and primers. Mice were maintained in both barrier and non-barrier facilities. The male mice were monitored weekly for unexpected weight gain and unusual behaviors. They were euthanized at the expected time points or when cachexia became inhumane. Tumors were harvested, weighed and measured using Vernier calipers. Tumor and control prostates were used in the immunohistochemistry (IHC) studies. Paraffin embedding and sectioning of mouse tissue followed by H&E histology was conducted with help from Dr. Brad Amendt's laboratory (University of Iowa). IHC was performed using the DAB150 IHC select® kit according to the manufacturer's protocol (EMD Millipore Inc). A PHF8 IHC-grade antibody (IHC-00343, Bethyl Labs) was used for staining. Images were examined and photographed using Nikon Eclipse 80i microscope and recorded with a Nikon digital camera.

The tissue array (TMA), which comprises a total of 41 PCa cases, was obtained from Vancouver Prostate Centre Tissue Bank. The radical prostatectomy samples collected from 20 untreated patients and 21 TURP specimens were collected from patients with CPRC. The control cores in this TMA are brain, glioma and small cell carcinoma of lung. The tissue microarray was manually constructed (Beecher Instruments, MD, USA) by punching duplicate cores of 1 mm for each sample. The H&E slides were reviewed and the desired areas were marked on them and the corresponding paraffin blocks. IHC staining was conducted using the Ventana autostainer model Discover XT™ (Ventana Medical System, Tuscan, Arizona), with an enzyme labeled biotin streptavidin system and solvent-resistant DAB Map kit, using rabbit polyclonal antibody against PHF8 at a concentration of 1/100 (IHC-00343, Bethyl Labs).

For digital Imaging and scoring, the stained TMA slide was digitalized with the SL801 autoloader and Leica SCN400 scanning system (Leica Microsystems; Concord, Ontario, Canada) at a magnification equivalent to X40. The images were subsequently stored in the SlidePath digital imaging hub (DIH; Leica Microsystems) of the Vancouver Prostate Centre. Values on a four-point scale were assigned to each immunostained sample. The following qualitative scheme was applied: 0 represents no staining of any tumor cells; 1 represents a faint staining or focal; 2 represents staining of convincing intensity in a minority of cells; and 3 represents staining of convincing intensity in a majority of cells. The staining intensity scores are presented as dot plots with Mean+/− Standard Deviation, and were generated using GraphPad Prism™. ANOVA and Student's T-test were used to infer statistical significance between PCa specimen groups.

### Bioinformatics and statistics

Expression data were inferred from GEO datasets GSE 39461, subseries GSE39452 [12] and GSE51463 [20]. GO TERM analyses used DAVID (https://david.ncifcrf.gov) [22]. All statistical analyses, including Student's T-Test, were performed on data obtained from at least three independent experiments. Results are expressed as the Mean ± SD.

## SUPPLEMENTARY FIGURES AND TABLES




