# Supplementary material for: c-MYC drives histone demethylase PHF8 during neuroendocrine differentiation and in castration-resistant prostate cancer
Source: Oncotarget. 2016 Sep 28;7(46):75585–602. doi: 10.18632/oncotarget.12310 (PMC5342763; doi:10.18632/oncotarget.12310)
Supplement: Supplementary file 1 [file oncotarget-07-75585-s001.pdf]

# c-MYC drives histone demethylase PHF8 during neuroendocrine differentiation and in castration-resistant prostate cancer

## SUPPLEMENTARY FIGURES AND TABLES

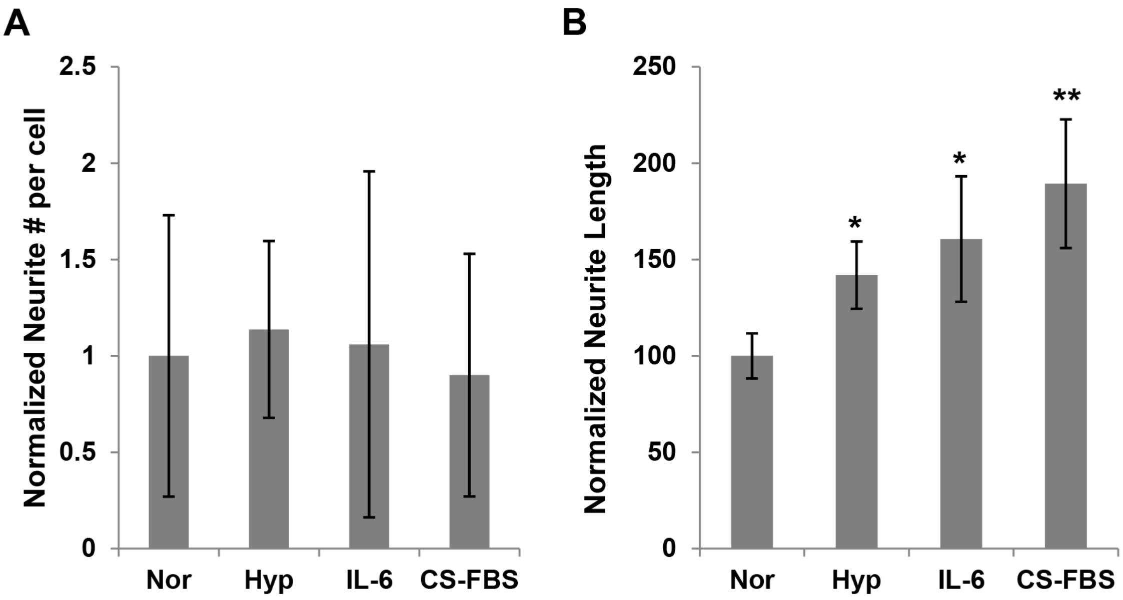


**Supplementary Figure S1: Six-day NED inductions extend neurite length in LNCaP cells. A.** Neurite number per cell were counted in 13-20 bright field images of LNCaP cells under normal conditions (Nor) or exposed to 1% hypoxia (Hyp), 20ng/ml IL-6 and CS-FBS for six days. **B.** Neurite length in cells treated as in A. was measured using Adobe Photoshop and normalized to normal condition. S.D. was obtained from all pooled measurements in each treatment. *: $p<0.05$.

**A**

| | hg19 position-minus strand | Ago2 ClipSeq Read Number in HeLa or 293* cells |
|---|---|---|
| hPHF8 3' UTR | chrX: 53963113-54071569 | |
| miR-22-3p | chrX: 53963744-53963765 | 92.452 |
| miR-9-5p | chrX: 53963983-53963989 | 16 |
| miR-9-5p | chrX: 53963549-53963555 | 12 |
| Let-7a-5p | chrX: 53963963-53963969 | 8 |
| let-7b-5p | chrX: 53963963-53963969 | 8 |
| miR-182-5p | chrX: 53965054-53965081 | 4.015* |
| miR-31-5p | chrX: 53965066-53965075 | 4.015* |

**B**

**miR-22-3p seed sequence: 1840-1847***

```
5' ...UCUUGAUCUUUCAAA-GGCAGCUA...
                 |||||  |||||||
3'       UGUCAAGAAGUUGACCGUCGAA
```

***Human PHF8 mRNA 3' UTR (2478bp, NM_015107)**

**C**

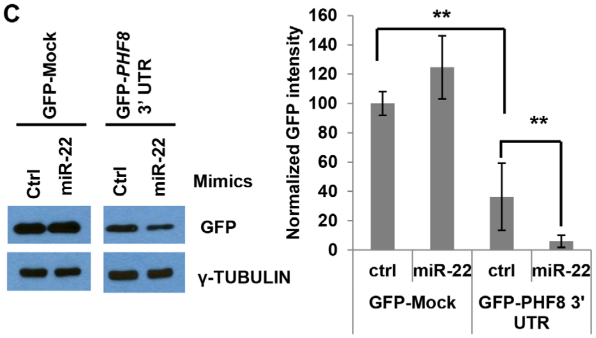


**D**

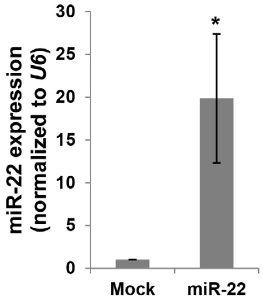


**Supplementary Figure S2: miR-22 targets and regulates *PHF8* 3' UTR. A.** Analysis of Ago2 CLIPseq data using starBase show potential binding sites of microRNAs on *PHF8* 3' UTR. **B**. The seed sequence of miR-22 at *PHF8* 3' UTR. **C**. LNCaP cell lines stably expressing pLenti-GFP-empty or pLenti-GFP-*PHF8* 3'UTR were transiently transfected with control or miR-22 mimics. GFP levels were assessed by immunoblotting (left panel) and quantified (right panel). **D**. The expression of miR-22 from the LNCaP stable cell lines overexpressing the GFP (mock) only or GFP-miR-22 constructs was assessed by RT-PCR. S.D. were obtained from three independent experiments. *: p<0.05; **: p<0.01.

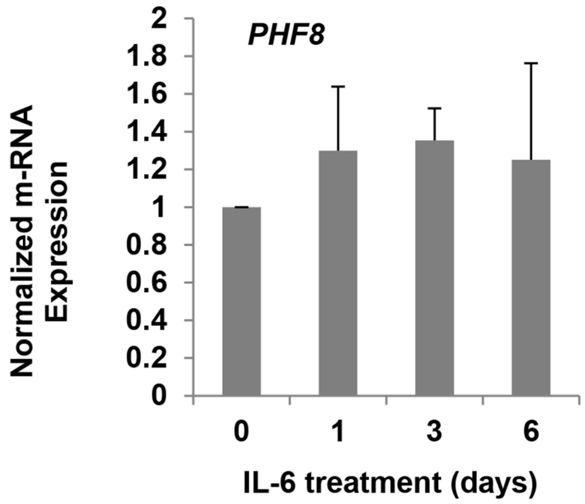


**Supplementary Figure S3: PHF8 is not transcriptionally regulated in the time course of IL-6 treatment in LNCaP cells.** PHF8 mRNA level was examined by RT-qPCR in LNCaP cells treated with IL-6 for the indicated time. S.D. were obtained from three independent experiments.

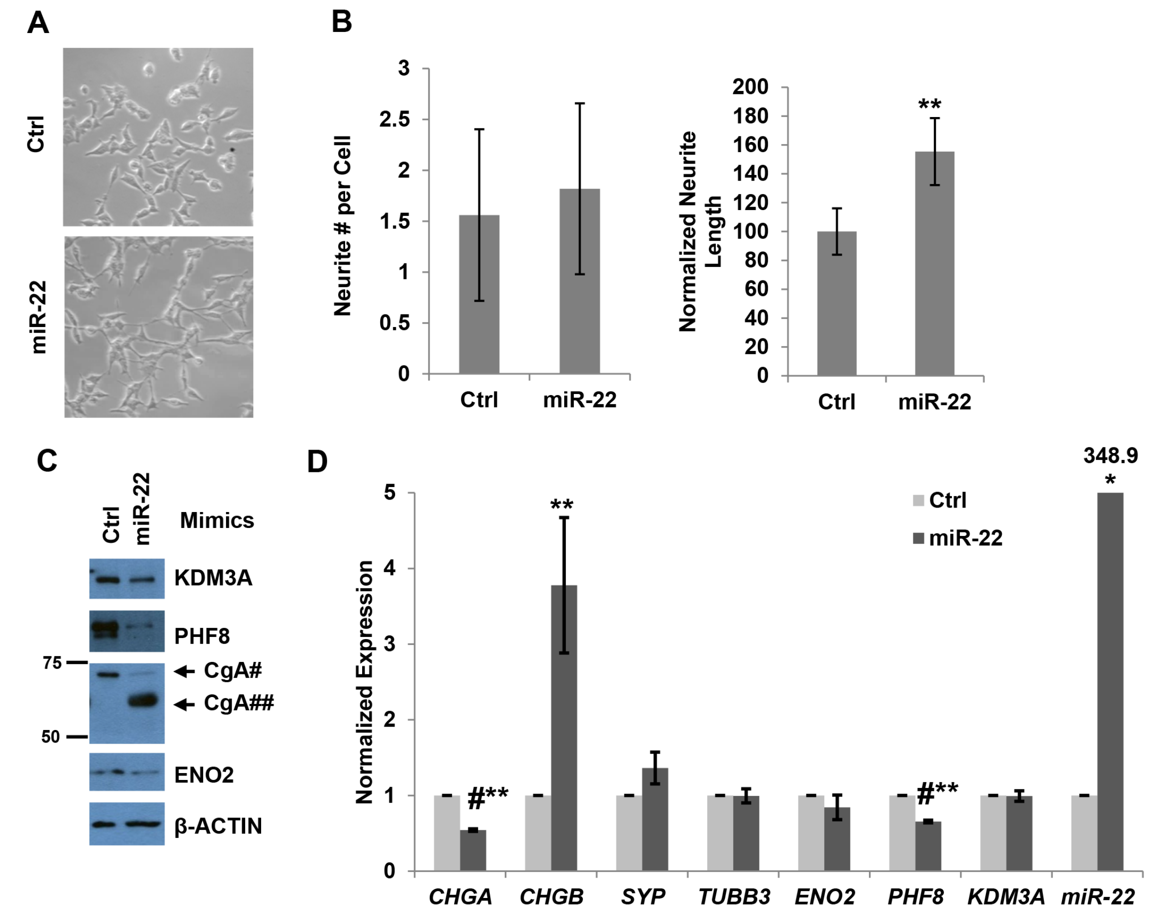


**Supplementary Figure S4: Transient transfection of miR-22 mimic in LNCaP cells induces partial NED features. A.** Representative images of 13 bright field images of LNCaP cells (total 63-72 cells) transiently transfected with control (Ctrl) or miR-22 mimics for 48 hours. **B**. Neurite number (left panel) and length per cell (right panel) were counted in the cells treated as in A. Quantification was performed using Adobe Photoshop and normalized to Ctrl. S.D. was obtained from all pooled measurements in each treatment. **: $p<0.01$. **C.** Indicated proteins from LNCaP cells treated as in A, as assessed by immunoblotting. #: precursor CgA, ##: intermediate CgA. **D**. RT-qPCR of indicated genes from cells cultured as in A. S.D. were obtained from at least three independent experiments. *: $p<0.05$; **: $p<0.01$; #: downregulation.

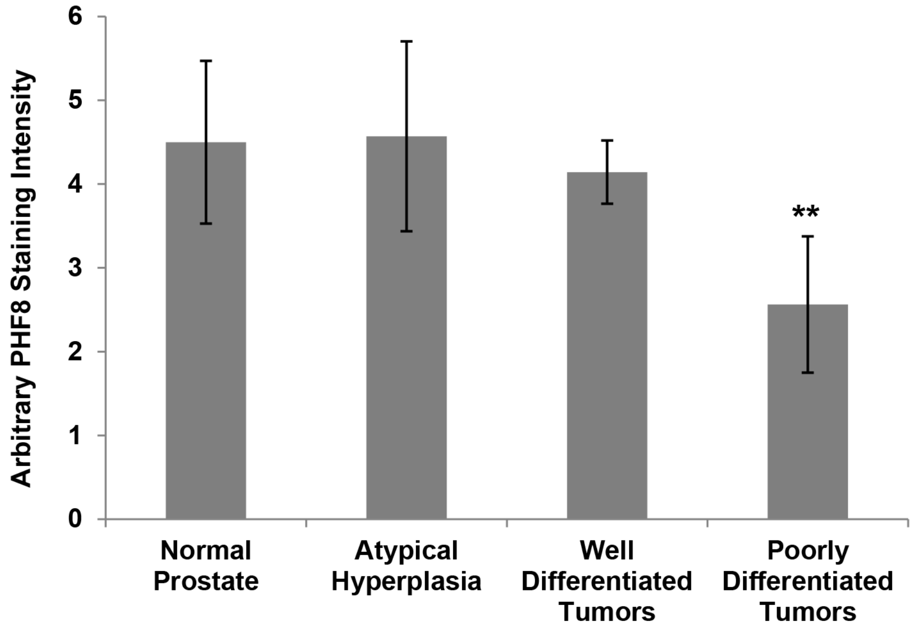


**Supplementary Figure S5: PHF8 expression decreases in poorly differentiated tumors from TRAMP mice.** PHF8 IHC staining was performed in normal prostate tissue (N=5) and tumors from TRAMP mice aged 3 to 5 months (N=7). PHF8 nuclear staining from non-redundant sections was scored between 1 and 5 (5 being highest intensity). PHF8 staining scores from atypical hyperplasia (7 sections), well differentiated (7 sections) and poorly differentiated (16 sections) tumors were compared with that from normal mouse prostate (10 sections). S.D. were obtained from each category. **: $p<0.01$.

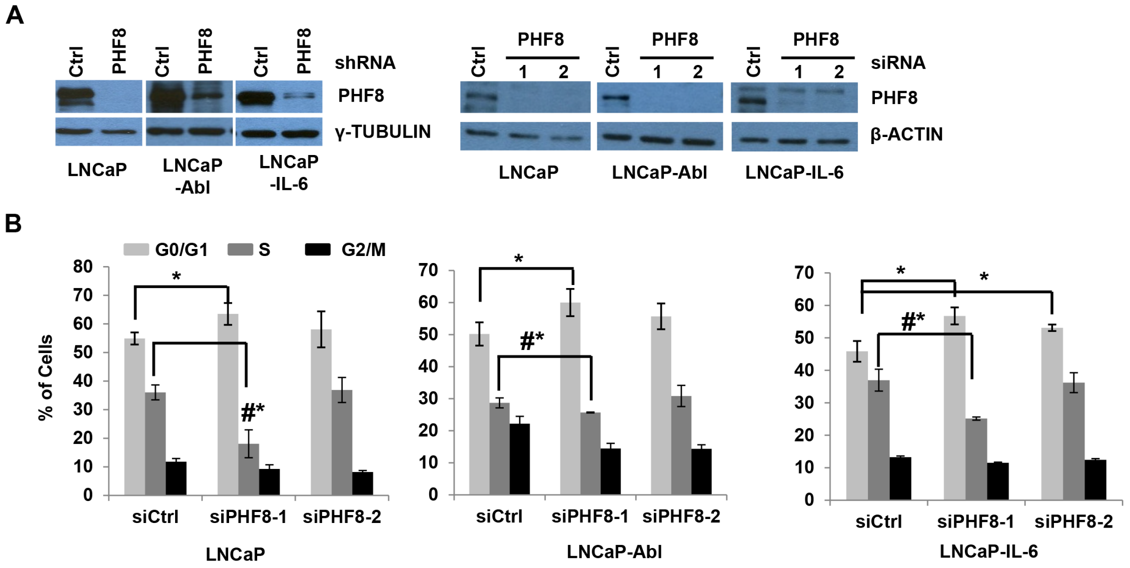


**Supplementary Figure S6: PHF8 knockdown accumulates G0/G1 cell cycle in LNCaP, LNCaP-Abl, and LNCaP-IL-6 cells. A.** PHF8 knockdown efficiency by doxycycline inducible shRNA and transient transfection of siRNAs was examined by western blotting. **B.** Cell numbers in each cell cycle phase in LNCaP, LNCaP-Abl and LNCaP-IL-6 cells transfected for 60 hours with scrambled siRNA (siCtrl) or two siRNAs targeting PHF8 (siPH8-1, -2). *: $p<0.05$; #: downregulation.

**Supplementary Table S1: RT-PCR primers**

| Genes | Primers | |
|---|---|---|
| KDM3A | Forward | GTCAACTGTGAGGAGATTCCAGC |
| | Reverse | AACTTCAACATGAATCAGTGACGG |
| PHF8 | Forward | GCCTTCTCCACTGAGGAGCAGGTA |
| | Reverse | CTCCTCATCCTGCCTTCCAGCTCT |
| KLK3 | Forward | TCTGCGGCGGTGTTCTG |
| | Reverse | GCCGACCCAGCAAGATCA |
| AR | Forward | TTGTCATGGAGCTGCAGATTCCA |
| | Reverse | TGCTGTCAGCATCCAAGTGGCT |
| SYP | Forward | ACCCACCTCCTTCTCCAATC |
| | Reverse | GATGAGGGGAGTGGAGTGAG |
| CHGA | Forward | GCGGTGGAAGAGCCATCAT |
| | Reverse | TCTGTGGCTTCACCACTTTTCTC |
| CHGB | Forward | CAGACTTCTATGATTCTGAGGA |
| | Reverse | CAGATCATGTGATGGCTGCT |
| ENO2 | Forward | AGCCTCTACGGGCATCTATGA |
| | Reverse | TTCTCAGTCCCATCCAACTCC |
| TUBB3 | Forward | GGCCTTTGGACATCTCTTC |
| | Reverse | TCGCAGTTTTCACACTCCTTC |
| c-MYC | Forward | TTCGGGTAGTGGAAAACCAG |
| | Reverse | CCTCCTCGTCGCAGTAGAAA |
| RPL13A | Forward | CCTGGAGGAGAAGAGGAAAGAGA |
| | Reverse | TTGAGGACCTCTGTGTATTTGTCAA |
| CCNA2 | Forward | CTGCATTTGGCTGTGAACTAC |
| | Reverse | ACAAACTCTGCTACTTCTGGG |
| CDK1 | Forward | GGCCAGAAGTGGAATCTTTACA |
| | Reverse | GGATCATAGATTAACATTTTCGAGAT |
| CDK4 | Forward | GAAACTCTGAAGCCGACCAG |
| | Reverse | AGGCAGAGATTCGCTTGTGT |
| UBE2C | Forward | CCCAACATTGATAGTCCCTTGA |
| | Reverse | AGAGTCCTATACAGAAATCACA |
| CDKN1A | Forward | CTGCCCAAGCTCTACCTTCC |
| | Reverse | CAGGTCCACATGGTCTTCCT |
| E2F2 | Forward | ACAAGGCCAACAAGAGGCTG |
| | Reverse | TCAGTCCTGTCGGGCACTTC |
| IL-6 | Forward | CCGCTTCGGCAGCACATATAC |
| | Reverse | TGACCAGAAGAAGGAATGCCCAT |
| U6 | Forward | CGCTTCGGCAGCACATATAC |
| | Reverse | AAAATATGGAACGCTTCACGA |
| Universal miR | Reverse | GTGCAGGGTCCGAGGT |
| miR-22 | Forward | GCGACAAGCTGCCAGTTGAA |
| | RT primer | GTCGTATCCAGTGCAGGGTCCGAG |
| | | GTATTCGCACTGGATACGACACAGTTC |

**Supplementary Table S2: Pattern 2. Genes up-regulated in LNCaP-DHT and down-regulated in LNCaP-Abl cells**

See Supplementary File 1
